# Role of Osteopathic Manipulative Treatment in Perioperative Pain Management: A Systematic Review With Exploratory Meta-Analysis

**DOI:** 10.7759/cureus.106138

**Published:** 2026-03-30

**Authors:** Kiran Ghotra, Aditi Iyer, Joshua Uberti, Nathan Armstrong, Shengyi Lai, Alex Whittaker, Tyler Scherer, Karthik Iyer

**Affiliations:** 1 Anesthesiology, Lake Erie College of Osteopathic Medicine, Erie, USA; 2 Internal Medicine, Lake Erie College of Osteopathic Medicine, Erie, USA; 3 General Surgery, Lake Erie College of Osteopathic Medicine, Erie, USA; 4 Critical Care Medicine, Mercy Hospital Jefferson, Festus, USA

**Keywords:** analgesic consumption, multimodal pain management, osteopathic manipulative treatment, pain reduction, perioperative pain

## Abstract

Postoperative pain remains a significant clinical challenge, and opioid-based analgesia alone is often insufficient, while also contributing to adverse outcomes and long-term opioid dependence. Osteopathic manipulative treatment (OMT) has been proposed as a nonpharmacologic adjunct within multimodal postoperative pain management frameworks, but the evidence has not been formally synthesized.

We searched PubMed, EMBASE, CINAHL, Scopus, and the Cochrane Central Register of Controlled Trials in January 2026, supplemented by a manual journal search of the Journal of the American Osteopathic Association, International Journal of Osteopathic Medicine, Journal of Osteopathic Medicine, and Journal of Manual & Manipulative Therapy. Eligible studies were randomized or matched controlled trials that evaluated OMT as an adjunctive intervention in postoperative patients and reported quantitative pain outcomes. Primary outcomes included pain intensity, analgesic consumption, functional recovery, and length of hospital stay. Secondary outcomes included respiratory function, patient satisfaction, and adverse events.

Three studies met the inclusion criteria and were carried forward to the final analysis. Two randomized controlled trials measuring postoperative pain via the Visual Analog Scale (VAS) demonstrated reductions of approximately 1.7 to 2.2 points in OMT-treated patients compared to controls at their respective assessment endpoints, exceeding established thresholds for clinical significance. A third prospective matched controlled study found that OMT patients achieved earlier functional milestones compared to controls, though differences in supplemental analgesic consumption were not statistically significant. An exploratory pooled analysis of the two VAS trials, using a DerSimonian-Laird random-effects model, yielded a mean difference of -1.16 (95% CI: -2.16 to -0.15); while this estimate excludes zero, with only two contributing studies and substantial heterogeneity (I² = 86.3%), it remains statistically fragile and should not be interpreted as confirmatory.

The narrative synthesis of individual trial results provides a more defensible basis for clinical inference at this stage: across independent controlled trials in cardiac and orthopedic surgical populations, OMT was consistently associated with reduced pain, lower analgesic requirements, and faster functional recovery. Formal meta-analytic conclusions are not warranted given the current evidence base. Larger multicenter trials with standardized protocols are needed to define OMT's role in perioperative care.

## Introduction and background

Postoperative pain remains a major challenge in surgical care. Despite real progress in anesthetic techniques and pain management strategies, most patients still experience moderate to severe pain after surgery, and the consequences of suboptimal pain control extend well beyond discomfort. With roughly 234 million major surgical procedures performed worldwide each year, inadequate pain management represents a widespread clinical problem [1].

The downstream effects of poorly managed postoperative pain are well documented. Uncontrolled pain impairs breathing, suppresses immune function, drives sympathetic activation, and restricts early mobilization, all of which raise the risk of pneumonia, cardiac ischemia, deep vein thrombosis, and pulmonary embolism [1,2]. In the longer term, inadequate pain control can lead to chronic postsurgical pain, prolonged opioid use, and reduced functional outcomes [3,4].

Opioids remain the backbone of postoperative analgesia, but their side effect profile is significant. Nausea, constipation, urinary retention, ileus, sedation, and respiratory depression are common enough to meaningfully delay recovery [5]. More concerning is the longer-term picture: perioperative opioid exposure is a recognized gateway to persistent use, even in opioid-naive patients. Studies have shown that 6%-10% of previously opioid-naive surgical patients develop persistent opioid use, and patients receiving an opioid prescription after short-stay surgeries face a 44% increased risk of long-term use [6,7]. This has pushed the field toward opioid-sparing multimodal frameworks, including Enhanced Recovery After Surgery (ERAS) pathways [8,9].

Osteopathic manipulative treatment (OMT) is one nonpharmacologic option within this multimodal framework. Grounded in osteopathic principles, OMT targets somatic dysfunctions, defined as impaired or altered function of the skeletal, arthrodial, and myofascial structures and their related vascular, lymphatic, and neural elements, that can perpetuate pain, restrict mobility, and disrupt normal physiology [10,11]. Techniques such as myofascial release, muscle energy, counterstrain, diaphragm doming, suboccipital inhibition, and lymphatic drainage work through overlapping mechanisms, modulating nociceptive input, improving lymphatic and circulatory flow, and rebalancing autonomic activity [12,13]. These mechanisms speak directly to the pathophysiology of the postoperative state, where inflammation, edema, biomechanical disruption, and autonomic dysregulation fuel pain and slow recovery [14].

A growing body of evidence across orthopedic, cardiothoracic, abdominal, and gynecologic surgical populations suggests that OMT can reduce postoperative pain, lower opioid requirements, support earlier ambulation, improve respiratory mechanics, and shorten hospital stays [15-17]. The safety profile, when OMT is applied by trained practitioners, appears favorable [18,19]. However, the literature remains limited and heterogeneous, and a formal synthesis has not yet been conducted.

This systematic review with exploratory meta-analysis aims to address that gap by evaluating OMT as an adjunctive intervention for postoperative pain management. Given the limited number of available controlled trials, we present a primary narrative synthesis of individual study findings alongside a pooled quantitative analysis, which should be interpreted as directionally informative rather than definitive. The goal is to situate OMT within the multimodal, opioid-sparing perioperative care model that the field is increasingly adopting [20].

## Review

Methods

Search Strategy

This systematic review and meta-analysis were conducted in accordance with PRISMA guidelines. A comprehensive literature search was conducted in January 2026 using PubMed, EMBASE, CINAHL, Scopus, and the Cochrane Central Register of Controlled Trials. This was supplemented by a manual search of relevant journals, including The Annals of Thoracic Surgery, Alternative Therapies in Health and Medicine, BMJ Open, Complementary Therapies in Medicine, the Journal of the American Osteopathic Association, JAMA Internal Medicine, and Integrative Cancer Therapies. The search was restricted to full-length, English-language studies, with no date restrictions applied.

In PubMed, the search was structured as: ("osteopathic manipulative treatment" OR "osteopathic manipulation" OR "OMT") AND ("postoperative" OR "perioperative" OR "post-surgical" OR "after surgery") AND ("pain" OR "analgesia" OR "analgesic") AND ("randomized controlled trial"[pt] OR "controlled trial"[pt] OR "prospective study"[pt]). Equivalent Boolean strings using MeSH terms and database-specific controlled vocabulary were applied in EMBASE, CINAHL, Scopus, and the Cochrane Register. In EMBASE, Emtree terms included 'osteopathic medicine'/exp, 'postoperative pain'/exp, and 'randomized controlled trial'/exp. In CINAHL, subject headings included 'Manipulation, Osteopathic' and 'Pain, Postoperative'. In Scopus and the Cochrane Register, free-text equivalents were applied with the same Boolean logic. Additional free-text variants included 'OMT cardiac surgery,' 'OMT orthopedic surgery,' 'OMT abdominal surgery,' 'OMT thoracic surgery,' and 'OMT analgesic use.'

Eligibility Criteria

To be included, a study had to meet three criteria: (1) be a randomized controlled trial or matched controlled trial; (2) evaluate OMT as an intervention in postoperative patients; and (3) report quantitative pain outcomes. Studies were excluded if they lacked quantitative pain data, focused on non-surgical pain, examined isolated techniques without a controlled comparator, were case reports or case series without controls, were published as abstracts only, or did not provide sufficient statistical data for analysis.

Study Selection

**Figure 1:**
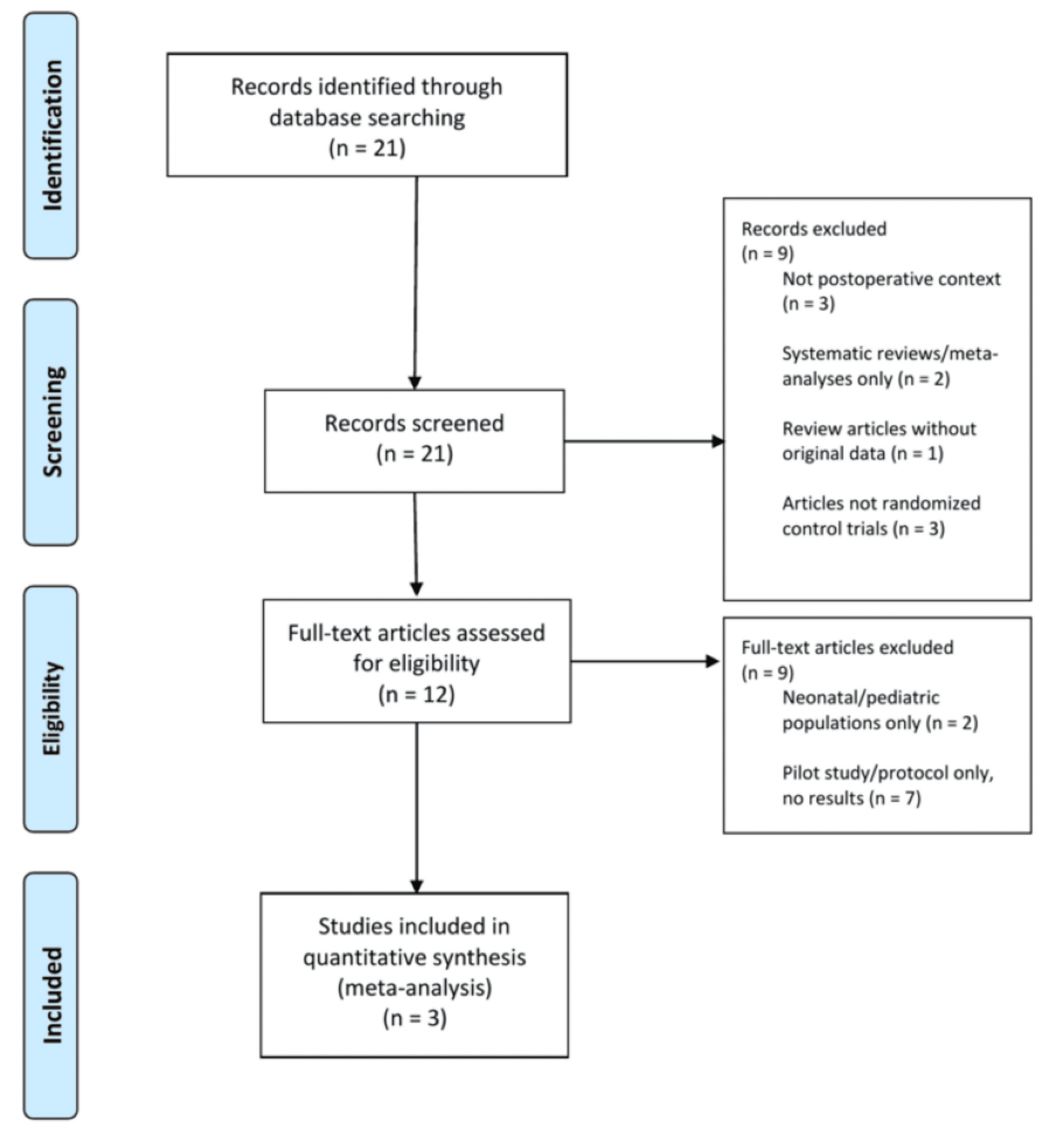
Flow Diagram of the Inclusion Criteria of Studies Eligible for Meta-Analysis

Twenty-one records were identified through database searching and manual journal review; no duplicates were identified. Two reviewers independently screened titles and abstracts. After title and abstract screening, nine records were excluded: three for not being in a postoperative context, two for being systematic reviews or meta-analyses only, one for being a review article without original data, and three for not being randomized controlled trials. Twelve full-text articles were assessed for eligibility; nine were further excluded (two for neonatal/pediatric populations only; seven for being pilot studies or protocols without reportable results). Three studies met all inclusion criteria and were carried forward to final analysis (Figure 1). Supporting claims were derived from peer-reviewed articles [1-31].

Risk of Bias Assessment

Formal risk of bias assessment was not conducted using a standardized tool (e.g., Cochrane RoB 2.0), which is acknowledged as a significant limitation of this review. Below we provide a narrative, domain-level appraisal of each included study across four domains: randomization and allocation concealment, blinding, completeness of outcome reporting, and overall bias judgment.

Racca et al. [18] reported randomization by computer-generated list with concealed allocation, which represents an adequate randomization procedure. Blinding of participants and practitioners was not feasible given the nature of the intervention; however, outcome assessors (a psychologist and physiotherapists) were described as blinded to group allocation. Outcome reporting appeared complete, with no obvious selective reporting. Overall risk of bias is assessed as moderate, primarily due to the open-label nature of the intervention.

Roncada [19] described random allocation using opaque sealed envelopes, with blinding of rehabilitation staff and outcome assessors to patient allocation; treating osteopaths alone were aware of group assignment. Follow-up was reported at 12 weeks and 1 year, with low apparent dropout, reducing concerns about attrition bias. Limited detail on sequence generation precludes a fully definitive selection bias assessment. Overall risk of bias is assessed as moderate.

Jarski et al. [24] used a prospective matched controlled design rather than true randomization, introducing potential for selection bias and confounding by indication. Outcome assessors (physical therapists evaluating ambulation and stair negotiation) were blinded to group assignment. The use of analgesic consumption and functional recovery as proxy pain outcomes, while clinically relevant, introduces additional confounding from institutional protocols and nursing practices. Overall risk of bias is assessed as moderate to high.

Data Extraction and Synthesis

Data were extracted using a standardized form covering: author and year, study design, sample size, surgical type, patient demographics, OMT protocol, control intervention, outcome measures, assessment timing, and statistical data, including means, standard deviations, confidence intervals, and p-values. Where studies reported medians and interquartile ranges (specifically Racca et al. [18], which reported median VAS scores with IQRs), values were converted to approximate means and standard deviations using the Wan et al. [31] method, as follows: $\mathrm{mean} \approx \frac{Q_1 + \mathrm{median} + Q_3}{3}$; $\mathrm{SD} \approx \frac{Q_3 - Q_1}{1.35}$. For Racca et al. [18], the OMT group at discharge yielded a converted mean of 1.33 (SD 0.74), and the control group a converted mean of 3.00 (SD 1.48). These conversions introduce a degree of imprecision that is reflected in sensitivity analyses. A DerSimonian-Laird random-effects meta-analysis was performed using the two studies that reported comparable VAS pain data; a random-effects model was selected a priori, given the anticipated clinical heterogeneity across surgical populations, intervention protocols, and outcome timing, with the high observed heterogeneity (I² = 86.3%) further supporting this choice. Because only two studies contributed to the pooled estimate, I² statistics should be interpreted with particular caution, as heterogeneity estimates are unstable with small numbers of studies. Assessment of small-study effects and publication bias via funnel plot or Egger's test was not feasible, given the inclusion of only two studies, which represents an additional limitation of the quantitative synthesis. 

Results

Three controlled trials examining adjunctive OMT in postoperative surgical patients were included (Table 1). All three used randomized or prospective controlled designs and evaluated OMT as an add-on to standard postoperative or rehabilitation care, with comparator groups receiving usual care alone. Pain outcomes were assessed either through validated pain scales or through analgesic use and functional recovery measures that serve as reasonable proxies for pain burden.

**Table 1:** Summary of Included Studies Note: In the Jarski et al. study [24], the reduction in supplemental intramuscular analgesic use in the OMT group (37%) compared to controls (50%) did not reach statistical significance (p > 0.05). OMT: osteopathic manipulative treatment; VAS: Visual Analog Scale; CABG: coronary artery bypass grafting; IM: intramuscular

Study	Surgery Type	N	OMT Intervention	Pain/Analgesic Outcomes	Functional Outcomes	Safety Profile
Jarski et al. (2000) [24]	Knee/Hip Arthroplasty	76 (38/group)	OMT on postoperative days 2-5	Fewer supplemental IM analgesics in the OMT group (days 2-5); between-group difference not statistically significant (p > 0.05)	20% faster stair negotiation (4.3 vs 5.4 days, p = 0.006); 43% greater ambulation on day 3 (24.3 m vs 13.9 m, p = 0.008)	No adverse events reported
Racca et al. (2017) [18]	Cardiac Surgery with Sternotomy	80 (40/group)	OMT + cardiorespiratory rehabilitation	VAS reduction: converted mean 1.33 vs 3.00 at discharge (MD ≈ -1.67, p < 0.01); analgesic drug intake similar between groups	27% higher inspiratory volume (1,781 vs 1,400 mL, p < 0.01); 12% shorter hospitalization (19.1 vs 21.7 days, p < 0.05)	No adverse events reported
Roncada (2020) [19]	CABG Surgery (Chronic Thoracic Pain)	308 (154/group)	OMT added to standard postoperative care	Mean VAS reduced from 3.6→0.80 at 12 wk and 3.6→0.56 at 52 wk vs control 2.6→1.2 (p = 0.030 at 12 wk; p = 0.014 at 52 wk)	Improved long-term thoracic mobility and functional tolerance associated with pain reduction	No adverse events reported

*Visual Analog Scale* (*VAS) Pain Outcomes*

Pain intensity was measured directly using the VAS in two of the three studies, Racca et al. [18] and Roncada [19] (Table 2). Because Racca et al. reported outcomes as medians with interquartile ranges rather than means and standard deviations, the Wan et al. [31] conversion method was applied prior to meta-analytic pooling (see Data Extraction and Synthesis). The converted mean VAS scores for Racca et al. at rehabilitation discharge were 1.33 (SD 0.74) for the OMT group and 3.00 (SD 1.48) for the control group, yielding an individual study mean difference of -1.67 (95% CI: -2.18 to -1.15). Roncada [19] reported mean VAS scores directly: 0.56 in the OMT group versus 1.20 in controls at 52 weeks, for a mean difference of -0.64 (95% CI: -1.18 to -0.10).

**Table 2:** Study Characteristics and Pain Outcomes of OMT vs Standard Care (VAS) Pain scores are reported using the VAS (0-10). *Denotes statistically significant mean difference. Pooled estimate derived from DerSimonian-Laird random-effects model. OMT group mean and SD for Racca et al. [18] are converted from median (1) and IQR (1-2) using Wan et al. [31]; control group values are converted from median (3) and IQR (2-4). Note: The fixed-effects model yielded a mean difference of -1.18 (95% CI -1.55 to -0.81). Given the substantial heterogeneity (I² = 86.3%), the DerSimonian-Laird random-effects model is reported as the primary pooled estimate. OMT: osteopathic manipulative treatment; VAS: Visual Analog Scale

Study	Surgery Type	OMT (n)	Control (n)	OMT Pain Score (VAS mean)	Control Pain Score (VAS mean)	Mean Difference (95% CI)	Weight (%)
Racca et al. (2017) [18]	Cardiac (sternotomy)	40	40	1.33 (SD 0.74) (converted from median)	3.00 (SD 1.48) (converted from median)	-1.67 (-2.18, -1.15)*	50.3
Roncada (2020) [19]	CABG (52 weeks)	154	154	0.56	1.20	-0.64 (-1.18, -0.10)*	49.7
Pooled Effect (random-effects)	-	194	194	-	-	-1.16 (-2.16, -0.15)	100

Roncada [19] conducted a randomized controlled trial examining chronic thoracic pain after coronary artery bypass grafting (CABG), with assessments at baseline, 12 weeks, and 1 year. At 12 weeks, the OMT group showed a substantially greater reduction in pain than controls: mean VAS scores dropped from 3.6 to 0.80 in the OMT group, compared with a decline from 2.6 to 1.2 in controls, a statistically significant difference (p = 0.030). That gap held at one year (p = 0.014), with OMT patients maintaining low pain levels, while the control group scores were essentially unchanged. Within-group reductions in the OMT arm were approximately 2.8 points at 12 weeks and just over 3 points at one year, well exceeding the 1.0-2.0 point threshold commonly cited as the minimum clinically important difference on the 0-10 VAS [21]. The persistence of effect at one year is clinically meaningful, given that chronic post-sternotomy pain affects 17%-56% of patients and represents a difficult-to-treat sequela for which pharmacologic options alone are often inadequate [22,23]. The 52-week time point was selected for pooling because it represents the final reported outcome and captures the durability of effect; sensitivity analyses using the 12-week endpoint are noted in the quantitative synthesis section.

Racca et al. [18] examined postoperative pain in older adults recovering from cardiac surgery via sternotomy during an inpatient cardiorespiratory rehabilitation program. Baseline pain was comparable between groups (median VAS 4 in both). By the time of rehabilitation discharge, patients in the OMT group reported median VAS scores near 1, versus near 3 in controls, a reduction of approximately 2 points in the OMT arm compared with approximately 1 point in controls. A change of 2 points or greater on the 0-10 VAS is generally considered a substantial clinical benefit [21].

Exploratory Meta-Analysis

**Figure 2:**
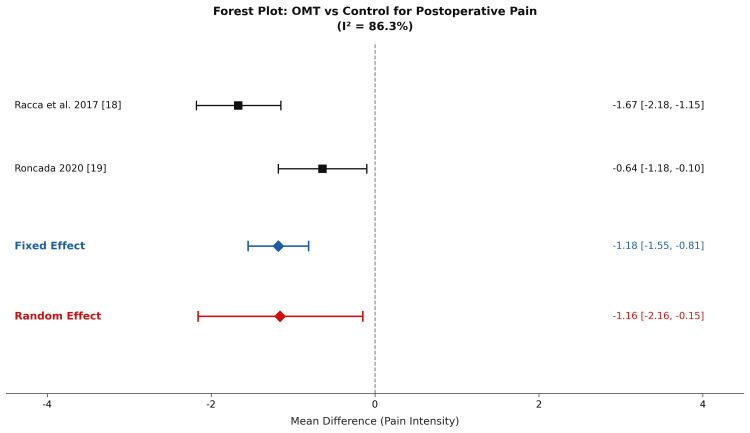
Forest Plot of the Effect of OMT on Postoperative Pain Intensity Compared With Standard Care in Patients Undergoing Cardiac Surgery Pain intensity was measured using the VAS (0-10, where 0 = no pain and 10 = worst pain imaginable). Included studies were Racca et al. [18] and Roncada [19]. Negative mean differences favor OMT. OMT: osteopathic manipulative treatment; VAS: Visual Analog Scale

A random-effects meta-analysis was performed using data from these two randomized controlled trials (Table 2). Using the DerSimonian-Laird estimator, the pooled mean difference was -1.16 (95% CI: -2.16 to -0.15), favoring OMT. Although the confidence interval excludes zero under the random-effects model, this estimate must be interpreted with caution, given the statistical fragility expected from a two-study pool. Between-study heterogeneity was substantial (I² = 86.3%, τ² = 0.45, χ² = 7.29, df = 1, p = 0.007), reflecting meaningful differences in patient population, timing of assessment (inpatient rehabilitation discharge vs. 52-week follow-up), and rehabilitation context. Because only two studies contributed to this pooled estimate, the I² statistic is unreliable, and effect estimates should be interpreted as directionally supportive rather than confirmatory (Figure 2).

Analgesic Consumption and Functional Recovery

The third study, Jarski et al. [24], took a different approach. This was a prospective matched controlled outcome study in patients undergoing major orthopedic procedures (knee or hip arthroplasty) that did not use a numerical pain scale. Instead, it tracked analgesic requirements and functional recovery as indicators of pain burden. Although more control group patients (50%, n = 19) required supplemental intramuscular analgesics on postoperative days 2 through 5, compared to OMT-treated patients (37%, n = 14), this between-group difference was not statistically significant. Functional recovery told a different story: OMT patients negotiated stairs earlier (4.3 vs 5.4 days, p = 0.006) and walked greater distances by postoperative day 3 (24.3 m vs 13.9 m, p = 0.008). Since early mobility after surgery is tightly linked to pain control, improvements here are consistent with reduced pain-related limitation during recovery, even in the absence of a statistically significant analgesic consumption difference.

Across all three studies, the pattern is directionally consistent: VAS reductions and faster functional recovery all favor OMT. Because only two studies reported comparable pain scale data, the pooled meta-analytic estimate should be viewed as a supplement to, rather than a replacement for, the narrative synthesis of individual trial findings presented above.

Discussion

Summary of Evidence

The findings across these three controlled trials point in a consistent direction: when OMT is added to standard postoperative or rehabilitation care, patients tend to experience meaningfully less pain and recover faster. The evidence base is small, but the consistency across different surgical populations makes a reasonable case for taking OMT seriously as a component of multimodal postoperative pain management. This is consistent with broader evidence supporting nonpharmacologic adjuncts in ERAS pathways, where the goal is adequate analgesia through multiple mechanisms, rather than reliance on any single agent [8,9,25].

In cardiac surgery patients specifically, the analgesic signal was strong. Both Roncada [19] and Racca et al. [18] reported VAS reductions in the 1.7- to 2.8-point range across individual study estimates, which clears the bar for clinical significance by most standards [21]. The durability of Roncada's findings at one year post-CABG is particularly notable, suggesting OMT may have a more lasting effect on pain trajectories than would be expected from a purely symptomatic intervention [19]. Racca et al. found that OMT patients experienced substantially greater pain reduction than controls during inpatient rehabilitation, despite comparable baseline scores, suggesting OMT may potentiate the pain-relieving effects of rehabilitation through improved thoracic mobility and modulation of autonomic tone [18].

Jarski et al. [24] did not measure pain directly, but provided supportive, indirect evidence through functional recovery measures; findings were broadly consistent with other controlled trials in orthopedic and abdominal surgical populations [15-17,26,27]. It should be noted that the reduction in supplemental analgesic use observed in the Jarski OMT group did not reach statistical significance, and this finding should not be characterized as a demonstrated reduction in analgesic consumption.

Mechanistic Considerations

The mechanisms behind these effects are not fully established, but are biologically plausible. OMT likely reduces nociceptive input by addressing somatic dysfunctions arising from surgical trauma, prolonged immobility, and altered biomechanics [10,11]. Modulation of sympathetic hyperactivity may also play a role, both in pain perception and in regional circulation and lymphatic drainage, supporting tissue healing [13,14]. Preliminary data from controlled trials suggest that OMT shifts autonomic balance toward greater parasympathetic tone, with measurable changes in heart rate variability, supporting this hypothesis [28].

Limitations

The limitations of this review are substantial and warrant explicit acknowledgment. First, only two studies contributed to the primary pooled pain outcome, yielding a combined sample of 388 patients in the VAS analysis (464 across all included studies). With so few studies, the I² statistic is unreliable as an estimate of true heterogeneity, and pooled effect estimates are correspondingly fragile. Second, the two VAS trials differ substantially in timing of outcome assessment: Racca et al. [18] measured pain at inpatient rehabilitation discharge (approximately three weeks post-surgery), while the Roncada [19] data used in the pooled analysis reflect 52-week outcomes; pooling across these timepoints is a meaningful source of heterogeneity. Third, outcome measures were not uniform across studies. Synthesizing direct pain scores with indirect indicators, such as analgesic use and functional recovery, requires caution. Fourth, a formal, study-level risk of bias assessment using a standardized tool (e.g., Cochrane RoB 2.0) was not conducted; the narrative domain-level appraisals provided represent an approximation rather than a rigorous bias evaluation. Fifth, expectancy effects cannot be fully excluded, though the magnitude and persistence of results in randomized designs make placebo alone an unlikely complete explanation. Sixth, all three included studies were conducted in cardiac or orthopedic surgical populations, limiting generalizability to other surgical contexts.

Given these limitations, the narrative synthesis of individual trial results provides a more defensible basis for clinical inference than the pooled meta-analytic estimate alone. The findings presented here should be treated as hypothesis-generating.

Implications for practice and future research

Despite these limitations, the consistent directional signal across independent trials suggests OMT warrants further investigation as a component of multimodal perioperative care. Any intervention that demonstrably reduces postoperative opioid exposure, while supporting recovery, is clinically relevant in the context of the ongoing opioid epidemic [29,30].

Future work should focus on larger multicenter randomized trials with standardized OMT protocols, consistent pain outcome measures (preferably means and standard deviations to facilitate future pooling), and longer follow-up periods. Formal risk of bias assessment, using validated tools (e.g., Cochrane RoB 2.0), should be incorporated into future systematic reviews. Systematic tracking of analgesic use across trials, with pre-specified statistical thresholds, would help clarify OMT's role within broader multimodal pain strategies. Head-to-head comparisons with other nonpharmacologic interventions, including transcutaneous electrical nerve stimulation, manual lymphatic drainage, and acupuncture, would also be informative for situating OMT within the broader landscape of integrative perioperative care.

## Conclusions

Postoperative pain is a genuinely difficult clinical problem, and pharmacologic approaches alone do not solve it for every patient. The evidence reviewed here provides a directional signal that OMT offers a meaningful adjunct: reductions in pain intensity and faster functional recovery, without adding medication-related risk. Rather than replacing standard analgesics, OMT appears to complement them by targeting biomechanical and neurophysiologic contributors to pain that opioids and nonsteroidal anti-inflammatory drugs (NSAIDs) do not directly address. The high between-study heterogeneity and the small number of included trials limit the confidence with which formal meta-analytic conclusions can be drawn, and a narrative synthesis of individual study results provides a more defensible clinical inference at this stage. Nevertheless, the consistency of positive outcomes across independent controlled trials is encouraging and justifies investment in the rigorous, standardized trials needed to settle the remaining questions.
